# A Toxicogenomic Approach for the Prediction of Murine Hepatocarcinogenesis Using Ensemble Feature Selection

**DOI:** 10.1371/journal.pone.0073938

**Published:** 2013-09-10

**Authors:** Johannes Eichner, Nadine Kossler, Clemens Wrzodek, Arno Kalkuhl, Dorthe Bach Toft, Nina Ostenfeldt, Virgile Richard, Andreas Zell

**Affiliations:** 1 Center for Bioinformatics Tuebingen (ZBIT), University of Tuebingen, Tübingen, Germany; 2 Nonclinical Drug Safety, Boehringer Ingelheim Pharma GmbH & Co. KG, Biberach an der Riss, Germany; 3 Nonclinical Drug Safety, H. Lundbeck A/S, Valby, Denmark; 4 Non-Clinical Development, UCB Pharma S.A., Brussels, Belgium; National Institute of Genomic Medicine, Mexico

## Abstract

The current strategy for identifying the carcinogenicity of drugs involves the 2-year bioassay in male and female rats and mice. As this assay is cost-intensive and time-consuming there is a high interest in developing approaches for the screening and prioritization of drug candidates in preclinical safety evaluations. Predictive models based on toxicogenomics investigations after short-term exposure have shown their potential for assessing the carcinogenic risk. In this study, we investigated a novel method for the evaluation of toxicogenomics data based on ensemble feature selection in conjunction with bootstrapping for the purpose to derive reproducible and characteristic multi-gene signatures. This method was evaluated on a microarray dataset containing global gene expression data from liver samples of both male and female mice. The dataset was generated by the IMI MARCAR consortium and included gene expression profiles of genotoxic and nongenotoxic hepatocarcinogens obtained after treatment of CD-1 mice for 3 or 14 days. We developed predictive models based on gene expression data of both sexes and the models were employed for predicting the carcinogenic class of diverse compounds. Comparing the predictivity of our multi-gene signatures against signatures from literature, we demonstrated that by incorporating our gene sets as features slightly higher accuracy is on average achieved by a representative set of state-of-the art supervised learning methods. The constructed models were also used for the classification of Cyproterone acetate (CPA), Wy-14643 (WY) and Thioacetamid (TAA), whose primary mechanism of carcinogenicity is controversially discussed. Based on the extracted mouse liver gene expression patterns, CPA would be predicted as a nongenotoxic compound. In contrast, both WY and TAA would be classified as genotoxic mouse hepatocarcinogens.

## Introduction

A crucial part of the drug development pipeline is the assessment of the carcinogenic potential of compounds, which is currently performed on the basis of the 2-year rodent bioassay. However, as these assays are associated with high costs as well as long study times, and require a high number of animals (more than 800 mice and rats) [1], there is a strong interest to develop supplementary approaches which facilitate drawing hypotheses about the carcinogenic risk of a compound at an earlier stage of the development process. Reliable predictive models of hepatocarcinogenesis could then be employed to increase the efficiency of the screening process by prioritization of drug candidates in the preclinical phase [2].

In general, carcinogens (C) can be subdivided into two groups depending on their mechanism. Genotoxic carcinogens (GC) are characterized by reactivity with DNA or by the formation of DNA-reactive metabolites which may lead to tumor initiation. Nongenotoxic carcinogens (NGC) do not directly cause DNA modifications, but mediate tumor initiation or promotion by secondary mechanisms, which are not completely discovered so far. In recent years, a wide variety of mechanisms causing nongenotoxic carcinogenesis in liver were proposed, such as oxidative stress, chronic cell injury, immunosuppression, induction of peroxisome proliferation, modulation of specific cytochrome P450 enzymes, increased secretion of growth-stimulating hormones, or the perturbance of specific signaling pathways [3], [4]. Ultimately, these mechanisms cause increased induction of mitosis, which is typically accompanied by decreased susceptibility to cellular mechanisms triggering apoptosis in degenerated cells [3], [4].

For the detection of GCs a battery of three *in vitro* genotoxicity tests (Ames test, mouse lymphoma assay, *in vitro* micronucleus or chromosomal aberrations test) is routinely applied during the early phase of drug development [5]. However, these short-term bioassays are insufficient in terms of specificity for the detection of NGCs, which are characterized by more complex mechanisms and thus require the performance of long-term tests.

Compounds such as TAA and WY which show a negative Ames test but a positive mouse lymphoma test are controversially classified by different authors as nongenotoxic or genotoxic carcinogens (reviewed by Waters *et al.*) [1]. Thus, it was suggested to classify these compounds as Ames-negative genotoxic compounds [1]. Especially for such compounds, which cannot definitely be assigned to either GCs or NGCs, approaches providing additional mechanistic insights would be beneficial during cancer risk assessment.

In more recently developed toxicogenomics approaches molecular events preceding neoplasia are considered to allow for the prediction of carcinogenicity based on characteristic gene expression patterns emerging after short-term exposure. These approaches typically employ microarray technology in conjunction with machine learning techniques for the early prediction of drug-induced carcinogenesis based on characteristic gene expression profiles [6]–[8]. A microarray-based approach also allows for drawing hypotheses about the mechanisms of genotoxic (GC) and nongenotoxic carcinogens (NGC) [9], [10]. However, in contrast to animal studies, toxicogenomics-based examinations of cancer risk are typically focused to specific target organs, such as, most notably, the liver.

In a pioneering study Nie *et al.* employed cDNA microarrays to profile mRNA expression in rat livers treated with 24 NGCs and 28 non-carcinogens (NCs) [11]. Combining the outcomes of diverse statistical and heuristic feature selection methods (e.g., *t*-test, genetic algorithms, etc.), a pooled gene list was generated, from which a short-list of 6 signature genes was extracted using an exhaustive enumeration method. More recently, Uehara *et al.* published a rat liver signature comprising 112 genes which was inferred using prediction analysis for microarrays (PAM) [12]. The signature was shown to facilitate the highly sensitive detection of carcinogenesis after single exposure, owing to a relatively high false positive rate. Inspired by the work of Auerbach *et al.,* who recommended longer exposure periods, another signature comprising 82 probesets was published by Uehara *et al.* [8]. Using this signature, a considerably lower fraction of false positives was observed at comparable sensitivity when performing Support Vector Machines-based NGC/NC classification of expression profiles resulting from 28 days of repeated dosing. As an aside, the informative probesets have also proven useful for the detection of GCs. For more detailed information about the designs of preceding studies, the profiled compounds and the estimated classification accuracies, the reader is referred to a comprehensive review by Waters *et al.* [1].

In contrast to the majority of recent toxicogenomics studies, which focused on elucidating NGC mechanisms in rat liver, Jonker *et al.* investigated the presence of characteristic, reproducible transcript expression patterns in diverse murine target organs [13]. The authors inferred organ-specific multi-gene biomarkers using supervised machine learning methods (e.g., Nearest Shrunken Centroids, Recursive Feature Elimination), which were also employed for the discrimination between GC, NGC and NC, either by solving one 3-class or two 2-class problems [13].

Here, we propose a new methodology, which employs ensemble feature selection in conjunction with bootstrapping for the extraction of mRNA signatures from transcriptomic profiles of GC-, NGC-, or NC-treated mice. Besides evaluating the average classification performance achieved by different algorithms for probeset selection, we also assess their robustness against small variations of the training data. When compared to recently published signatures for the prediction of hepatocarcinogenicity in mice, we found that our signatures are equally good or slightly better, depending on the employed classifier and evaluated prediction task.

In contrast to previous toxicogenomics studies, we profiled global gene expression in both male and female mice, which is also done on a routinely basis in chronic toxicity and carcinogenicity studies. As some of the general pathways and biological processes leading to cancer (e.g., cell proliferation) may be influenced by androgens and estrogens, we consider it relevant to include both sexes in the development of biomarkers for hepatocarcinogenesis. Ultimately, this study design facilitated capturing characteristic changes in gene expression, which are present in both sexes upon treatment with GCs and NGCs, respectively.

## Methods

### Ethics Statement

The animal experimental work under this study was subject to the Danish Executive Order No. 1306 of 23 November 2007. The protocol was reviewed by the veterinarians responsible for animal welfare in the testing facility and conducted under animal license number 2009.561.1593 issued by the Danish Animal Inspectorate. For necropsy the animals were anaesthetized in isoflurane and exsanguinated from the heart. All animals were checked for mortality/ill health twice daily and animals in extremis were sacrificed after consultation with the veterinarian in charge of animal health and welfare. All animal procedures described in the present paper have been approved by the ethical committee for animal experimentation of UCB Pharma S.A. and were in accordance with the most recent European and Belgian legislation on the use of laboratory animals (Directive 2010/63/EU, Belgian Royal Decree of 29 May 2013) as well as with the Guide for the Care and Use of Laboratory Animals (NRC, 2010).

### Animal Study

In this study, we employed the Affymetrix GeneChip Mouse Genome 430 2.0 Array to monitor the changes in the gene expression pattern induced by diverse compounds in mouse liver samples. The generated microarray data were deposited at Gene Expression Omnibus (GEO accession: GSE44783). Treatment groups of 6 male and female CD-1® mice, respectively, were dosed by oral gavage with a heterogeneous set of compounds over a time period of 3 or 14 days. Time matched control groups were treated with the corresponding vehicles, carboxymethyl cellulose or corn oil. The compounds, their CAS numbers, vehicles, doses and the group ID are all listed in Table 1. Dose levels were chosen on the basis of literature data, toxicological databases (CPDB database: http://potency.berkeley.edu/) [14], or dose range-finding studies. For the non-carcinogenic compounds a high pharmacological dose was selected. 24 hours after the last dose, the mice were anaesthetized in isoflurane, exsanguinated from the heart and subjected to necropsy. The left lateral liver lobe was cut in cubes with about 4–5 mm side length, placed in Wheaton Cryovials, snap frozen in liquid nitrogen and kept at −80°C until extraction. A histopathological examination was conducted for all liver samples from this study (data not shown).

**Table 1:** Overview of compounds used for training and evaluation of classifiers.

Class	Compound (Short name)	CAS Number	Vehicle	Dose[mg/kg/d]	Group ID (Dosingperiod of 3 days)	Group ID (Dosingperiod of 14 days)
**Genotoxic** **carcinogens (GC)**	C.I Direct Black (CIDB)	1937-37-7	CO	2500	CIDB_G67F_D4	CIDB_G69F_D15
					CIDB_G67M_D4	CIDBG69M_D15
	Dimethylnitro-samine (DMN)	62-75-9	CO	2	DMN_G70F_D4	DMN_G72F_D15
					DMN_G70M_D4	DMN_G72M_D15
	Methylen-dianiline (MDA)	101-77-9	CO	50	MDA_G86F_D4	MDA_G88F_D15
					MDA_G86M_D4	MDA_G88M_D15
				75	MDA_G87F_D4	MDA_G89F_D15
					MDA_G87M_D4	MDA_G89M_D15
**Undefined** **compounds**	Cyproterone acetate (CPA)	427-51-0	CO	160	CPA_G78F_D4	CPA_G80F_D15
					CPA_G78M_D4	CPA_G80M_D15
	Thioacetamid (TAA)	62-55-5	CMC	20	TAA_G57F_D4	TAA_G59F_D15
					TAA_G57M_D4	TAA_G59M_D15
	Wy-14643 (WY)	50892-23-4	CMC	200	WY_G52F_D4	WY_G54F_D15
					WY_G52M_D4	WY_G54M_D15
**Nongenotoxic** **carcinogens (NGC)**	1,4-Dichloro-benzene (DCB)	106-46-7	CO	600	DCB_G83F_D4	DCB_G85F_D15
					DCB_G83M_D4	DCB_G85M_G15
	Phenobarbital sodium (PB)	57-30-7	CMC	80	PB_G60F_D4	PB_G62F_D15
					PB_G60M_D4	PB_G62M_D15
	Piperonyl-butoxide (PBO)	51-03-6	CMC	600	PBO_G48F_D4	PBO_G50F_D15
					PBO_G48M_D4	PBO_G50M_D15
**Non-carcinogens** **(NC)**	Cefuroxime sodium (CFX)	56238-63-2	CMC	250	CFX_G25F_D4	CFX_G27F_D15
					CFX_G25M_D4	CFX_G27M_D15
	Nifedipine (Nif)	21829-25-4	CMC	50	Nif_G29F_D4	Nif_G31F_D15
					Nif_G29M_D4	Nif_G31M_D15
	Prazosin hydrochloride (Praz)	19237-84-4	CMC	5	Praz_G37F_D4	Praz_G39F_D15
					Praz_G37M_D4	Praz_G39M_D15
	Propranolol hydrocholride (Prop)	318-98-9	CMC	80	Prop_G32F_D4	Prop_G34F_D15
					Prop_G32M_D4	Prop_G34M_D15

The table lists all compounds along with their carcinogenicity class and CAS Registry Number. Corn oil (CO) or 0.1% carboxymethyl cellulose (CMC) was used as vehicle. Doses were selected for each compound based on the tumorigenic dose rate 50 (TD_50_), long-term animal studies leading to liver cancer known from the literature or initial dose range-finding studies. After a dosing period of 3 or 14 days the mouse livers were subjected to gene expression analysis using an Affymetrix platform. Two treatment groups, each comprising 5–6 male and female mice, respectively, were examined for each compound, dose and point of time. Time matched control groups were included for both vehicles. Each treatment group has a unique group ID which is composed of the compound short name, the group number and the sex (Female or Male).

### Tissue Sampling and Microarray Hybridization

Samples from 5–6 animals per treatment group were subjected to mRNA expression analysis. The snap-frozen liver samples were homogenized with Qiazol (Qiagen, Hilden, Germany). The cRNA synthesis, target hybridization, probe array washing, staining and subsequent probe array scanning were done according to the standard protocol 3′IVT Express Kit User Manual (Affymetrix). Microarray analysis was performed by using GeneChip Mouse Genome 430 2.0 Arrays from Affymetrix.

### Preprocessing of Microarray Data

After importing the Affymetrix raw data (i.e., CEL files) into the R programming language and environment for statistical computing, different metrics and statistics implemented in the package *arrayQualityMetrics* were used to assess the quality of the raw data [15]. As no major experimental problems could be detected, all arrays were included in further analysis steps. The raw data were then normalized using the RMA method and probesets were mapped to gene symbols and Entrez IDs using the appropriate metadata packages deposited at Bioconductor [16], [17].

### Analysis Pipeline for Signature Inference and Compound Classification

One of the main research objectives of this study was the selection of mRNA signatures, i.e., sets of informative marker genes, which are consistently differentially expressed between compound classes, and thus can be utilized to reliably predict the type of carcinogenicity of a given compound. For this purpose, we implemented an analysis pipeline for the inference and evaluation of gene signatures from mRNA expression data. The central element of this pipeline is a two-stage method, encompassing statistical and supervised learning methods for marker gene selection (Stage 1) and expression profile-based compound classification (Stage 2).

#### Stage 1: Extraction of gene signatures

In the first stage, we employed linear Support Vector Machines (SVM), SVM-based Recursive Feature Elimination (SVM-RFE), Prediction Analysis for Microarrays (PAM) and a signal-to-noise ratio (Golub-Ratio) proposed by Golub *et al.*, which measures the correlation between gene expression states and compound class labels [18]–[21]. All probesets represented on the microarray were considered as candidate markers and no filtering was performed based on differential gene expression. The marker genes selected should fulfill two requirements. Firstly, the signature probesets should be reliable, i.e., facilitate the accurate discrimination of the compound classes. Secondly, the signature should be robust, i.e., be largely independent of the employed selection algorithm and the set of samples used for training.

For optimization of the classification performance, we used a grid search approach to tune the individual parameters of the machine learning methods. For this purpose, we employed a 3×3 nested cross-validation procedure, which ensures unbiased parameter tuning, while maintaining independent test sets. The robustness of the signature was increased by performing ensemble feature selection with *m* = 4 methods on *n* = 25 randomly drawn bootstraps, each containing 90% of the training samples. For each bootstrap the remaining 10% of the training samples (*out-of-bag* samples) were used to assess the classification performance, achieved with the selected marker genes, in terms of area under the Receiver Operating Characteristic (ROC) curve.

In order to determine the optimal number of informative genes, the ROC scores were computed for *k* = 10 different probeset numbers between 2 and 100. Subsequently, the optimal number of features corresponding to the analytical maximum area under the ROC was estimated by fitting splines (implementation from package *splines* for R).

Next, for each feature selection method the robustness of the selected signatures, i.e., the correspondence of the informative gene sets inferred on different bootstraps, was measured based on the Kuncheva stability index [22]. The Kuncheva index (KI) is a measure for the consistency between multiple subsets of features, typically extracted from different bootstraps or by using different methods. In contrast to other stability indices, the KI also accounts for common selection of features purely by chance. It monotonically increases with the size of the intersection of the two compared subsets and is defined on a scale between −1 and 1. The value −1 is returned for disjoint subsets, each containing 50% of the features. 0 is expected for independently drawn subsets and 1 for identical subsets. Given a feature set *X* with *|X| = n* and two feature subsets *A*, *B* with *0<|A| = |B| = k<n* and *|A ∩ B| = r* the Kuncheva index is defined as 
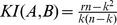
 [22]. KI can also be generalized to a score for measuring the stability of more than two selected subsets, simply by computing the mean of the KIs that have been computed for all pairs of subsets.

On the basis of the signatures inferred on different bootstraps using different methods, we generated consensus signatures by computing an average rank for each gene. For this purpose a consensus ranking was generated by computing the rank sums across all methods and bootstraps. Subsequently, the genes were sorted in ascending order by the rank sums.

#### Stage 2: Prediction of compound classes

In the second stage, the signatures inferred on the training data were incorporated as predictive features for compound class prediction by diverse classifiers, namely SVM, *k*-Nearest Neighbor (KNN), PAM, Naïve Bayes, Random Forest, and Weighted Voting [18], [20], [21], [23]. To this end, a stratified and nested 3×3 cross-validation was performed. While the algorithm-specific parameters were tuned in the inner 3-fold cross-validation, the performance was assessed based on the area under the ROC curve in the outer 3-fold cross-validation. The fold-changes of the signature genes obtained from Stage 1 were standardized by computing *z*-scores 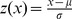 and then incorporated as features. In order to ease the interpretability of the classification outcomes, all prediction scores were transformed to a scale ranging from 0 to 1. SVM outputs (ranging between approx. −10 and 10) were rescaled using the function 
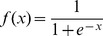
. Weighted Voting scores (originally between −1 and 1) were transformed using 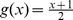 as scaling function. In order to obtain confidence values for KNN, the fraction of positively labeled neighbors among all nearest neighbors was calculated. All other methods inherently produce prediction scores between 0 and 1. The employed classification methods were either implemented from scratch in R or imported from the existing R packages *knnflex*, *pamr*, *klaR* and *randomForest* [20], [23]. For SVM classifiers we used implementations from the SHOGUN toolbox [18].

## Results

### Inference of Signatures for Early Prediction of Hepatocarcinogenesis in Mouse

**Figure 1:**
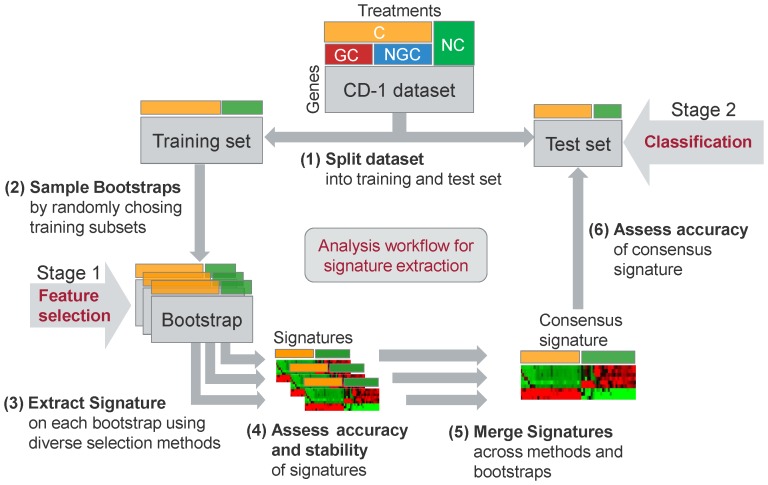
Overview of analysis workflow used for signature extraction and class prediction. The methodology used for the extraction of gene expression signatures is illustrated using our analysis of the CD-1 mouse dataset as example. This dataset contains mRNA expression data from male and female mice treated with GC, NGC, and NC. In the example a signature for the discrimination of C from NC is predicted using the analysis workflow shown above. (**1**) A stratified cross-validation is performed on the whole dataset. In each cross-validation fold the dataset is split into a training set and a test set. (**2**) *n = 25* bootstraps, each containing 90% of the training samples, are randomly chosen from the training data. (**3**) A signature, i.e., a subset of informative genes for compound class discrimination is selected on each bootstrap by using *m* = 4 different methods. (**4**) The mean classification performance achieved by each gene selection method is computed on the 10% *out-of-bag* samples, which are left out for each bootstrap. The stability of the consensus signature is assessed by computing the Kuncheva index from the list of signatures extracted from different bootstraps. (**5**) The *n×m* signatures selected by *m* methods on *n* bootstraps are merged, based on a joint ranking computed from the sums of the ranks calculated for individual signatures. (**6**) Various machine learning algorithms are employed to predict the carcinogenic compound class, based on the inferred consensus signature. The performance is evaluated on the independent test set and assessed in terms of area under the ROC curve.

Using an ensemble of statistical and machine learning-based feature selection techniques in conjunction with state-of-the-art methods for supervised classification, we constructed accurate and robust models for carcinogenic compound class prediction based on characteristic gene expression profiles. Our analysis workflow (Figure 1) was employed for the identification of multi-gene mRNA signatures for the discrimination of different compound classes (C: GC+NGC vs. NC, GC vs. NGC, GC vs. NC, and NGC vs. NC), which were profiled in CD-1 mice at two dosing times (3 days and 14 days) using an Affymetrix platform. The corresponding gene lists which were used to predict the carcinogenic outcome in both sexes are attached in Table S1.

**Figure 2:**
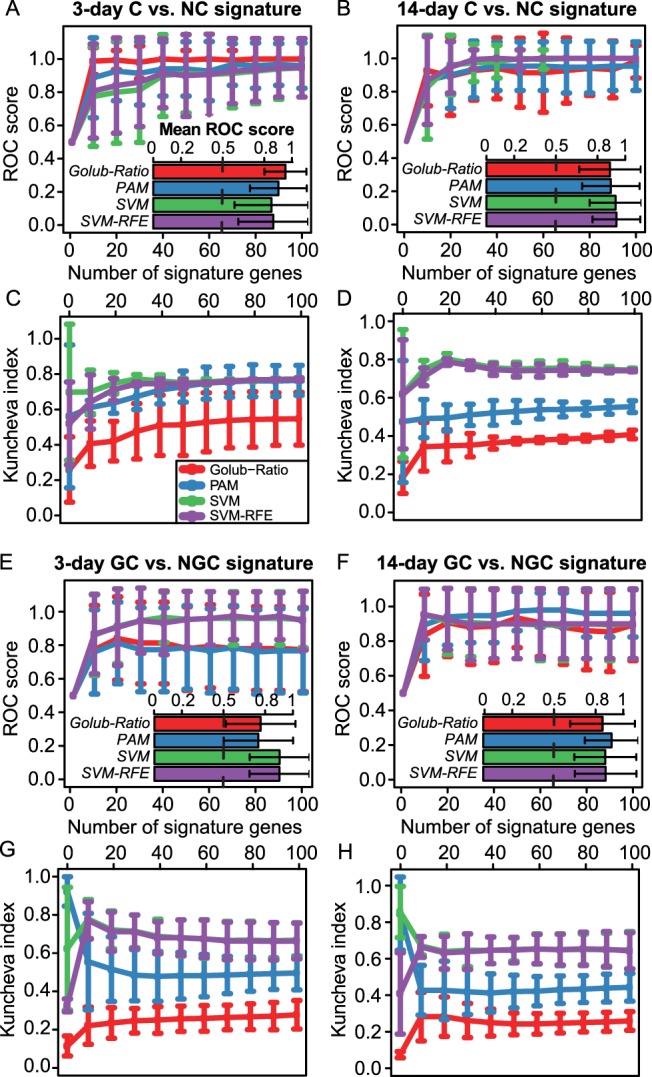
Accuracy and stability of signatures for compound classification. (**A**) The line plots depict the mean performance of C vs. NC classification after 3 days of repeated dosing, which was achieved based on gene sets extracted with different feature selection methods. Each curve corresponds to a feature selection method and the performance was assessed depending on the number of genes selected as informative features. The prediction accuracy was assessed on the samples left out from 25 random subsamplings of the dataset (bootstraps), each containing 90% of the data, and measured in terms of area under the ROC curve. The inset bar plot depicts the ROC scores averaged across bootstraps and signature sizes. (**B**) Performance of C vs. NC classification after 14 days of treatment illustrated as in (A). (**C**) The correspondence of the extracted C vs. NC gene sets across 25 bootstraps was assessed based on the Kuncheva stability index (KI) for each of the 4 employed feature selection methods. The KI was then for each method plotted against the number of selected signature genes. (**D**) Robustness of signatures for C vs. NC classification after 14 days of treatment illustrated as in (C). (**E, F**) Prediction accuracy achieved with signatures for GC vs. NGC classification after (E) 3 days and (F) 14 days of repeated dosing, respectively, depicted as in (A). (**G, H**) Similar illustration as in (C) showing robustness of signatures for GC vs. NGC classification after (G) 3 days and (H) 14 days of administration, respectively.

The ROC scores plotted for varying signature sizes in Figure 2A demonstrate that all tested feature selection methods are well suited to produce signatures for the early discrimination of C from NC, which are both accurate (ROC scores >0.81) and stable (max. KI ≈ 0.7), when compared to a random guesser (ROC = 0.5 and KI = 0). In comparison to the 3-day signature (Figure 2A), a slightly higher mean classification accuracy (ROC scores >0.84) and comparable stability was achieved by using the 14-day signatures (Figure 2B). This gain on performance is even more striking when comparing 3-day signatures and 14-day signatures for GC vs. NGC classification (Figure 2E–F). In summary, the performance of the individual methods heavily depends on the classification task, but only the SVM and SVM-RFE method consistently achieved classification accuracies >0.8 on all four datasets (Figure 2A,B,E,F). Furthermore, the two SVM-based methods were found to provide by far the most robust signatures, when assessing the correspondence of informative gene lists selected on different bootstraps, based on the stability index proposed by Kuncheva *et al.* (Figure 2C,D,G,H) [22].

The corresponding ROC and KI plots which resulted from the evaluation of signatures for GC vs. NC and NGC vs. NC classification, respectively, are shown in Figure S1.

**Figure 3:**
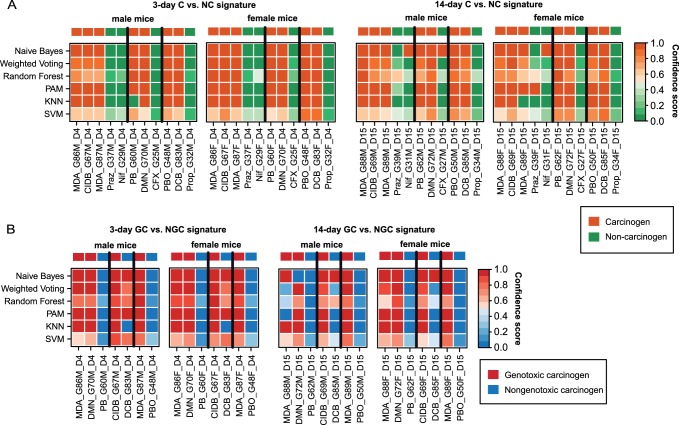
Classification results obtained for different signatures. (**A**) The four heatmaps show the predictions resulting from diverse binary classifiers for the discrimination of C from NC after 3 days (left two heatmaps) or 14 days (right two heatmaps) of repeated dosing. For each dosing time two heatmaps are depicted which correspond to male and female mice, respectively. The rows correspond to different classifiers and the columns to different treatment groups. The continuous prediction scores, returned from the classifiers, were transformed to confidence scores between 0 and 1, which provide an estimate of the probability of class C. The colorbar on top shows the true class annotation. The black vertical lines separate the test samples from the three folds of cross-validation. (**B**) Heatmaps illustrating the classification outcome of diverse predictors to distinguish GC from NGC based on characteristic gene expression profiles observed in male and female mouse liver samples after 3 or 14 days of administration. KNN, K-Nearest Neighbor; SVM, Support Vector Machine; PAM, Prediction Analysis for Microarrays.

On the basis of the signatures inferred from multiple bootstraps using different methods, we generated a consensus signature for each classification task as described in more detail in the methods section (Figure 1). For each classification task we trained six different classifiers (SVM, KNN, PAM, Random Forest, Weighted Voting, and Naïve Bayes) and evaluated their classification accuracy based on cross-validation. In order to ease the interpretation of the classification results, the outputs were transformed into confidence scores between 0 and 1, which serve as an estimate of the probability of the positive class. These confidence scores are illustrated as heatmaps in Figure 3. For most classifiers a high agreement was found between the prediction scores and the true class labels. This observation is consistent with the outcomes of ROC evaluation, where the average AUC ranged between 0.92 and 1.0 for C vs. NC classification and between 0.83 and 1.0 for GC vs. NGC classification. From Figure 3 it becomes apparent that almost perfect compound classification could have been achieved by a majority vote across all methods. Only for 1,4-dichlorobenzene (DCB) a reliable classification as either GC or NGC was not possible after 3 days of repeated dosing. However, after 14 days the compound was correctly classified as NGC by the majority of classifiers. The heatmaps showing the correspondence between the predicted and true classes obtained for GC vs. NC and NGC vs. NC classification are depicted in Figure S2.

We also evaluated the performance of two variants of our methodology for signature extraction. The first one exclusively adopts the two feature selection methods SVM and SVM-RFE, as for the corresponding signatures a consistently high performance and increased robustness across bootstraps was observed (Figure 2). Since a good separation of the compound classes could also be achieved by Principal Component Analysis (PCA), we employed PCA and the algorithmically related Partial Least Squares Discriminant Analysis (PLS-DA) for feature selection in a second approach. The gene rankings were derived from the absolute values of the loadings of the first principal and PLS component, respectively. Consistent with our expectations the SVM- and SVM-RFE-based signatures performed particularly well in conjunction with SVM classifiers in all four evaluated settings, i.e., C vs. NC and GC vs. NGC classification after 3 or 14 days (Figure S3). For the informative gene sets derived from PCA and PLS-DA slightly weaker performance was observed for C vs. NC classification, while GCs could be discriminated from NGCs with comparable accuracy. A comparison of the informative gene sets obtained from the different approaches showed that many genes were selected in common, which was especially the case for mRNA signatures used to discriminate GCs from NGCs (Figure S3). The mRNA signatures, which were compiled based on the two variants of our methodology, are available from Table S2 and S3.

### Classification of Compounds with Undefined Class Assignment

**Figure 4:**
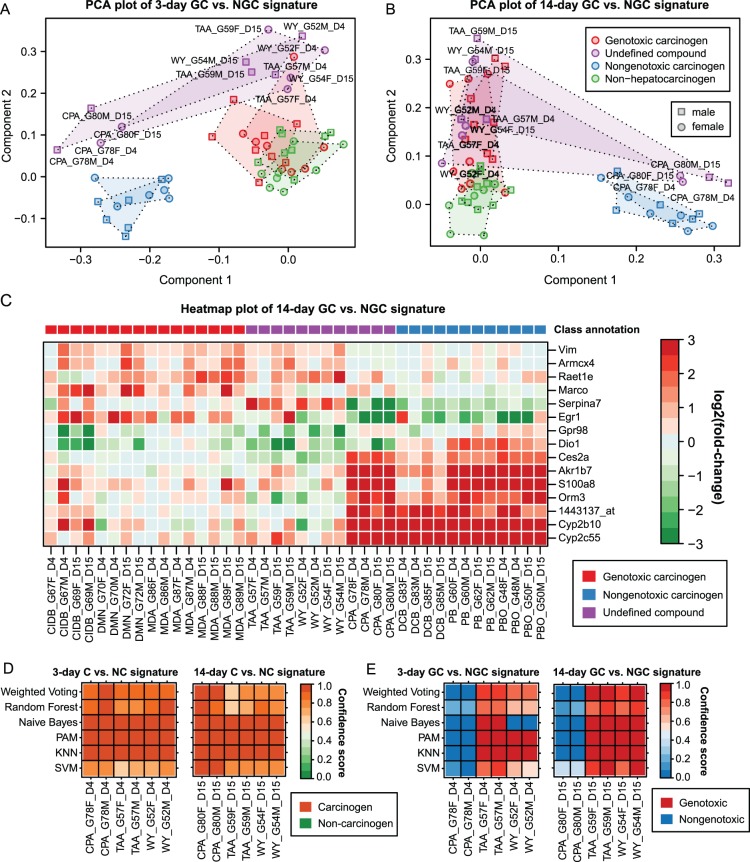
Reclassification of the compounds CPA, TAA, and WY. (**A**) Points indicate treatment groups which were originally represented by a vector containing the fold-changes of all signature genes and then transformed to its two principal components. Here, the informative genes for discriminating GC vs. NGC after 3 days of repeated dosing were used. Groups of male animals are drawn as squares and female ones as circles. The fill color of the points indicates the compound class. Polygons indicate the convex hulls of clusters corresponding to either male or female mice treated with a certain class of compounds. (**B**) Similar plot as in (A), but the multi-gene signature for GC vs. NGC classification after 14 days was used here. (**C**) The heatmap provides a graphical representation of the fold-changes of 15 selected signature genes from the 14-day signature for GC vs. NGC classification. Rows correspond to genes and columns to treatment groups. Upregulated genes are colored in red and downregulated ones in green. The colorbar on top indicates the corresponding compound classes. (**D**) Heatmaps showing confidence of predictions made by diverse C vs. NC classifiers for male (M) and female (F) mice treated with CPA, TAA and WY for 3 days (left heatmap) or 14 days (right heatmap). (**E**) Similar illustration as in (D) showing prediction outcomes of GC vs. NGC classifiers.

The classification of the test compounds TAA, WY and CPA with respect to their mechanism of rat liver cancer induction are controversially discussed [1]. In this study, these compounds were regarded as undefined chemicals, due to ambiguous results in genotoxicity testing. Consequently, these compounds were not used in the training set. Aiming at a classification of these compounds based on their gene expression profiles, we adopted different supervised classification methods, incorporating the informative genes from the analysis of the other compounds as features (see Figure 4D–E). Furthermore, we performed principal component analyses in order to visually inspect if the undefined compounds cluster in the vicinity of confidently annotated GCs or NGCs. For this purpose, each treatment group was represented by a vector containing the fold-changes of a certain multi-gene signature and then transformed to a two-dimensional space spanned by the two principal components explaining the highest fraction of the variance in the data.

It becomes obvious from the PCA plot of the 3-day GC vs. NGC signature that CPA clusters near the NGC compounds, whereas the points corresponding to TAA and WY are located in the vicinity of the GC cluster (Figure 4A). Strikingly, a clear separation of the compounds can be observed for both treatment durations and both sexes in the PCA plot of the 3-day as well as of the 14-day signature (Figure 4A–B). The differences in the gene expression patterns resulting from CPA vs. TAA or WY treatment also become apparent from the heatmap plot shown in Figure 4C. These findings are also consistent with the classification results obtained from diverse supervised learning algorithms (Figure 4D–E). Specifically, we found that the carcinogenic potential of the three “undefined” compounds was recognized by all six classifiers and except for Naïve Bayes, all methods classified TAA and WY as a GC and CPA as an NGC.

Furthermore, it is remarkable that the treatment-specific differences in the gene expression patterns observed between GC and NGC are considerably higher than the sex-specific differences observed between male and female mice (Figure 4A–C). This finding indicates that the selected informative genes are more strongly related to different mechanisms of hepatocarcinogenesis than to sex-specific physiological differences.

### Comparison with Signatures from Related Studies

In order to compare our signatures to those reported from previous studies, we assessed the prediction accuracy of diverse classifiers, incorporating either our novel signatures or known ones, based on ROC evaluation. To this end, we extracted three signatures for the early detection of hepatocarcinogenicity in mice from the literature (Table S4). Two of them were reported by Jonker *et al.,* who inferred one signature for separating C and NC and one for discriminating GC from NGC using a two-step strategy similar to our approach [13]. Another hepatocarcinogen-specific signature was more recently reported by Park *et al.,* who employed the Ingenuity Pathway Analysis software and demonstrated by hierarchical clustering analysis that their informative genes differ between GC and NGC [24].

**Figure 5:**
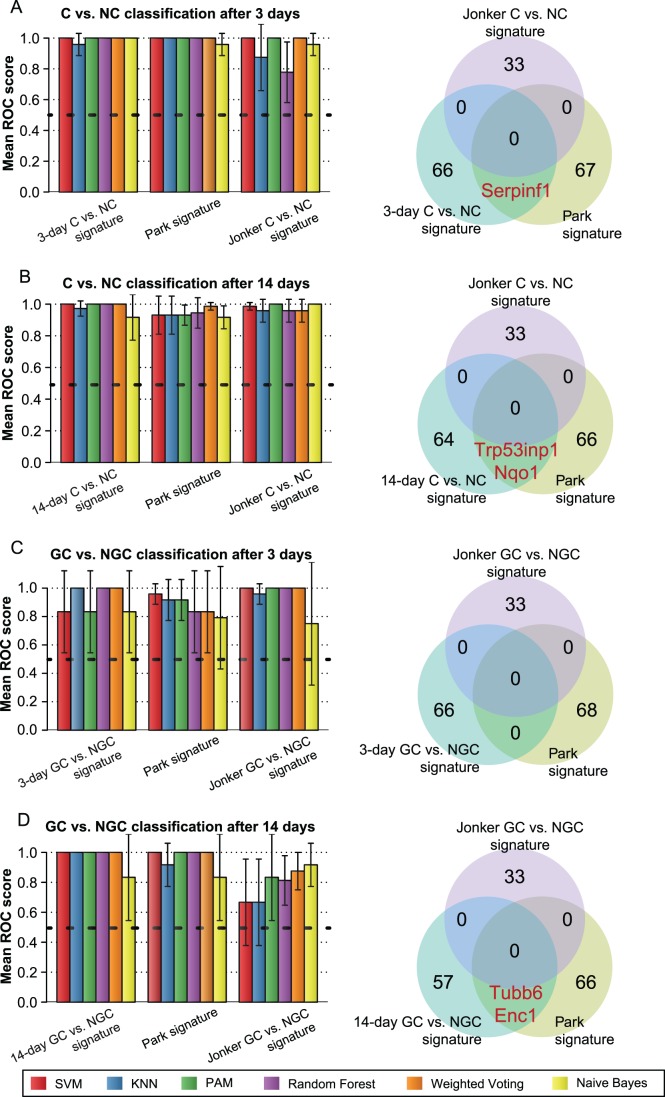
Performance comparison with signatures known from the literature. (**A**) The grouped bar plots depict the area under the ROC curves obtained for novel and known signatures for the separation of C and NC after 3 days of treatment. For this purpose, the performance of diverse classifiers was evaluated by a 3-fold cross-validation. Each bar corresponds to a certain classifier (see legend) and each group of bars refers to a certain signature. The horizontal dashed line indicates the performance that would have been achieved by random guessing. The adjacent Venn diagrams illustrate informative genes common between signatures. (**B**) Same plots as in (A), but for C vs. NC classification after 14 days of repeated dosing. (**C**) Mean ROC scores and signature overlaps for GC vs. NGC classification after 3 days of treatment. (**D**) Same plots as in (C), but for GC vs. NGC classification after 14 days of repeated dosing.

The results shown in Figure 5 indicate that all signatures achieve a considerably higher performance than a random guessing approach. However, only for our signatures, we found that the error-free prediction of compound classes (ROC score = 1) can be achieved in all classification tasks if either Random Forest or Weighted Voting is used. Except for the GC vs. NGC classification after 3 days of exposure, which appears to be the most challenging classification task, an error-free classification outcome could also be obtained from the SVM or PAM approach. Independent of the classification task a comparatively low average accuracy was observed for the Naïve Bayes method. In summary, the evaluation results suggest that our proposed mRNA signatures provide a valuable alternative to previously published gene sets, as in all four different settings equal or higher classification performance could be achieved (Figure 5A–D).

Comparing our sets of informative genes to previously compiled sets of potential marker genes, we found small overlaps between our signatures and those proposed by Park *et al*. (see Venn diagrams in Figure 5). The two signatures selected by Jonker *et al*. were disjoint with both our gene sets and the one by Park *et al*. [24]. The complementarity of the signatures proposed by Jonker *et al.* can in part be explained by the fact that the authors used a custom oligonucleotide array limited to 8205 probes, while a standard Affymetrix platform was used in both our study and the one conducted by Park *et al.* [24].

## Discussion

In this study we conceived, implemented and applied a novel ensemble feature selection approach for the extraction of robust multi-gene signatures from mRNA transcript expression data, which facilitates reliable compound classification for hepatocarcinogens. We analyzed an Affymetrix dataset obtained from male and female CD-1 mouse liver samples taken after treatment of mice for 3 or 14 days with diverse compounds (3 GC, 3 NGC and 4 NC).

In Figure 4C our most informative genes showing characteristic profiles upon treatment with genotoxic or nongenotoxic compounds are depicted. Within the European IMI MARCAR project (www.imi-marcar.eu), we are currently assessing the mechanistic relevance of such genes, which are specifically deregulated upon treatment with GCs and NGCs in order to gain a more profound understanding of the induced cancer types. Among the probesets which were specifically deregulated upon treatment with GCs and showed either inverse or unaltered patterns for NGCs were, for instance, *Vim*, *Armcx4*, *Raet1e*, *Marco*, *SerpinA7*, *Egr1* and *Gpr98*. In response to nongenotoxic carcinogenic exposure an upregulation of *Dio1*, *Ces2a*, *Akr1b7*, *S100a8*, *Orm3*, *Cyp2b10* and *Cyp2c55* was measured (Figure 4C).

Among others, the aforementioned probesets were used for the toxicogenomics-based examination of the carcinogenic class of the compounds TAA, WY and CPA. We considered these three substances as “undefined”, as it cannot be reliably determined, based on mutagenicity testing results, whether rodent liver tumors are induced by these compounds via a genotoxic or nongenotoxic mechanism. Detailed literature search concerning mutagenic effects of TAA revealed ambiguous results. While no genotoxicity was observed in the Ames test, commonly used genotoxicity tests such as the mouse lymphoma assay and the *in vivo* micronucleus test show both positive as well as negative results [25]–[27]. Another example for such an undefined compound is WY, which is in general classified as a peroxisome proliferator belonging to nongenotoxic carcinogens, whereas a single cell gel electrophoresis assay indicated induction of DNA damage in WY treated cells [28]–[30]. A third compound we classified as an undefined agent is CPA, which was negative in the Ames test [31], too, but showed positive results concerning mutagenicity in an *in vivo* liver micronucleus test in female rats [32].

A central aim of the here proposed ensemble feature selection method was the identification of class-specific mRNA signatures which allow for a proper compound classification. The informative probesets were then used to classify the three “undefined” substances based on their gene expression patterns. Concerning the signature genes, the expression patterns of TAA and WY resembled those of GCs, whereas the pattern observed for CPA was similar to that of NGCs (Figure 4E). Consistent with these findings, it was also suggested by Waters *et al.* to classify TAA and WY as Ames test negative genotoxic compounds [1].

In contrast to previous approaches, we employed ensemble feature selection which is uncommon in toxicogenomics, but has already been successfully used for other applications, such as cancer diagnostics and biomolecular text mining [33], [34]. We chose this emerging method to increase the stability and generalizability of the extracted mRNA signatures. Ideally, the extracted signatures should be robust against changes in the training data and independent of the applied feature selection method. Thus, we propose to generate a consensus signature derived from multiple bootstraps and an ensemble of algorithms for the selection of informative genes. To this end, we employed statistical filter methods as well as supervised wrapper methods for feature selection. While the latter ones involve the use of machine learning-based classifiers to assess the prediction accuracy for a selected subset of features, the former ones assess the relevance of features based on intrinsic properties of the data [35]. Especially wrapper methods which involve learning an abstract model from a set of training compounds may suffer from overfitting as the number of features (i.e., all probesets represented on the microarray) is typically much larger than the number of profiled compounds in toxicogenomics datasets. However, in this study all constructed models were found to be well generalizable to the unknown test compounds which is supported by a high performance in cross-validation.

Evaluating our predictive multi-gene biomarkers against signatures known from the literature based on cross-validation, we found that our mRNA signatures facilitate compound classification with comparable or higher accuracy depending on the classification task (Figure 5). Arguably, the literature-based signatures were not perfectly suited for all evaluated settings, as different mouse strains, treatment durations, compounds, and doses were used. Furthermore, while all published informative gene sets were derived from male mice exclusively, animals of both sexes were considered in our study for signature inference and evaluation. However, despite the different study designs, the performance comparison showed that the previously proposed signatures are fairly well generalizable.

In this study we demonstrated that despite of the additional variation in gene expression profiles caused by the inclusion of both sexes, we were able to extract robust and predictive gene sets which show characteristic patterns indicative of carcinogenesis irrespective of the sex of the sample material. We consider this as an advantage over currently available mRNA signatures for rodent hepatocarcinogenesis, which were derived solely from male animals, and hence may have an increased risk to fail in predicting female-specific carcinogens.

The choice of the right dose is crucial for the selection of signature genes which are specifically deregulated in response to the application of carcinogenic substances. Overdosing compounds may mask the specific changes in gene expression which are related to cancer initiation or progression, due to a huge amount of deregulated genes orchestrating an unspecific stress response [13]. On the other hand, low doses may be not tumorigenic, and hence not cause differential expression of potential biomarker genes. Jonker *et al.* proposed to use the maximum tolerated dose (MTD) for dosing, which can be determined in a subchronic study [13]. As for the compounds analyzed here chronic mouse studies have already been performed, we considered the cancerogenic dose from published animal long-term studies and a multiple of the TD_50_ rate, which induced tumors in half of the tested animals.

In a recent study, Auerbach *et al.* reported that the treatment time is also a critical factor, which may highly impact the success of a toxicogenomics approach [36]. The authors observed the lowest error and a considerable increase of SVM margin scores for their 90-day data, which points to a higher prediction confidence for long treatment durations [36]. Uehara *et al.* also reported that a higher accuracy of the prediction models could be achieved for longer exposure durations [8], [36]. Consistent with these findings our results indicate that after 14 days of repeated dosing a more reliable discrimination of GCs from NGCs is possible than after 3 days (Figure 3B). This applies in particular to the compound DCB, which was not correctly classified by the majority of classifiers after 3 days. The misclassification of DCB may be in part explained by deviations in its expression profile from the ones observed for other NGCs (Figure 4C). However, characteristic changes in gene expression, facilitating accurate detection of carcinogens in general could already be extracted after 3 days of treatment (Figure 3A). As the time of emergence of characteristic profiles may vary in a compound-specific manner, Ellinger-Ziegelbauer *et al.* proposed to represent each compound by expression data from multiple time points, at which a sufficient number of deregulated genes could be observed [6]. Nevertheless, we chose to focus on single time points, as the premature determination of the optimal time points may not be possible in the real use case, which is the classification of completely unknown compounds. Furthermore, as early cellular defense response patterns (e.g., enzyme induction) are expected to become apparent after 3 days of carcinogenic treatment, whereas adaptive changes in gene expression may be present in response to oxidative damage, cell death, persistent toxicity or inflammation after 14 days, we did not combine data from the two time points.

When assessing prediction accuracy depending on the signature size, we found that predictions with tolerable reliability are possible, based on the expression status of ten informative genes (Figure 2A–B, E-F). This finding is also supported by recent work from Uehara *et al.*, who published a signature consisting of 9 Affymetrix probesets (7 different genes), which was shown to discriminate NGC from NC in rat liver with reasonable accuracy [8].

Pathway enrichment analysis of the 66 genes from the 14-day C vs. NC signature (Table S1) against the KEGG database resulted in significant enrichments in the PPAR signaling pathway (p = 0.039) and the TGF-beta signaling pathway (p = 4.27E-4), which are both involved in known mechanisms of hepatocarcinogenesis [37], [38]. For both GC vs. NGC signatures, we observed a significant overrepresentation among genes related to metabolism of drugs and xenobiotics by cytochrome P450 (p<0.05).

In order to identify informative genes which are relevant for several classification tasks (C vs. NC, GC vs. NGC, GC vs. NC, NGC vs. NC) and show class-specific expression patterns at multiple time points (3 days, 14 days), we determined for each gene in how many of the 8 evaluated settings it was selected as an informative gene (Figure S4). The most frequently selected probesets present in more than 50% of the predicted signatures were *Cyp2c50*, *Cyp2c55*, *Cyp2b10*, *S100a8*, *Gsta1*, and *Akr1b7* (Figure S5). However, our results indicate that a more reliable classification can be achieved, if additional informative genes are taken into account.

As the susceptibility to carcinogenic compounds as well as the triggered changes in gene expression may vary between mouse strains, future analysis should be conducted in order to validate to what extent the inferred gene expression signatures can be reproduced in other strains. Especially, the applicability of our proposed signatures to transgenic mouse strains should be assessed in future studies, as for instance *Xpa/p53+/−* knockout mice were shown to improve the prediction accuracy of bioassays while at the same time reducing the required treatment duration [39]. Furthermore, we are currently assessing the correspondence between signatures inferred for mice and rat, respectively, in a closely related toxicogenomics study.

Recent toxicogenomics studies are mostly focused on profiling the RNA expression of rodents after single or repeated exposure to carcinogens [1]. Besides profiling the transcriptome, future studies should also investigate compound-induced changes in the DNA methylome, proteome or phosphoproteome, which could reveal novel biomarker candidates facilitating reliable classification of compounds with respect to their carcinogenic potential and mechanism.

As a wide variety of mechanisms was proposed for NGC, it is hardly feasible to extract a highly specific and at the same time universal signature capturing the gene expression changes caused by NGC treatment [3], [4]. However, public toxicogenomics databases (e.g., TG-GATEs and Drug Matrix), covering a rich set of compounds and multiple representatives of each NGC subclass, may facilitate the inference of mechanism-specific signatures which would firstly allow for a more fine-grained classification and secondly provide in-depth insights into individual mechanisms [40].

In conclusion, the here proposed methodology was shown to facilitate the extraction of robust gene expression signatures which were incorporated into generalizable models for the prediction of the carcinogenic class of compounds. These prediction models may accelerate the screening for promising candidates, and hence increase the efficiency and cost-effectiveness of preclinical drug development.

## Supporting Information

Figure S1 **Accuracy and stability of signatures for compound classification. (A)** The line plots depict the mean performance of GC vs. NC classification after 3 days of repeated dosing, which was achieved based on gene sets extracted with different feature selection methods. Each curve corresponds to a feature selection method and the performance was assessed depending on the number of genes selected as informative features. The prediction accuracy was assessed on the samples left out from 25 random subsamplings of the dataset (bootstraps), each containing 90% of the data, and measured in terms of area under the ROC curve. The inset bar plot depicts the ROC scores averaged across bootstraps and signature sizes. **(B)** Performance of GC vs. NC classification after 14 days of treatment illustrated as in (A). **(C)** The correspondence of the extracted GC vs. NC gene sets across 25 bootstraps was assessed based on the Kuncheva stability index (KI) for each of the 4 employed feature selection methods. The KI was then for each method plotted against the number of selected signature genes. **(D)** Robustness of signatures for GC vs. NC classification after 14 days of treatment illustrated as in (C). **(E, F)** Prediction accuracy achieved with signatures for NGC vs. NC classification after (E) 3 days and (F) 14 days of repeated dosing, respectively, depicted as in (A). **(G, H)** Similar illustration as in (C) showing robustness of signatures for NGC vs. NC classification after (G) 3 days and (H) 14 days of administration, respectively.
(PDF)

Figure S2 **Classification results obtained for different signatures. (A)** The four heatmaps show the predictions resulting from diverse binary classifiers for the discrimination of GC from NC after 3 days (left two heatmaps) or 14 days (right two heatmaps) of repeated dosing. For each dosing time two heatmaps are depicted which correspond to male and female mice, respectively. The rows correspond to different classifiers and the columns to different treatment groups. The continuous prediction scores, returned from the classifiers, were transformed to confidence scores between 0 and 1, which provide an estimate of the probability of class GC. The colorbar on top shows the true class annotation. The black vertical lines separate the test samples from the three folds of cross-validation. **(B)** Heatmaps illustrating the classification outcome of diverse predictors to distinguish NGC from NC based on characteristic gene expression profiles observed in male and female mouse liver samples after 3 or 14 days of administration. KNN, K-Nearest Neighbor; SVM, Support Vector Machine; PAM, Prediction Analysis for Microarrays.
(PDF)

Figure S3 **Classification results obtained for different ensembles of feature selection methods. (A)** The grouped bar plots depict the area under the ROC curves achieved with signatures which were inferred based on three different ensembles of feature selection methods and applied for the separation of C and NC after 3 days of treatment. The first ensemble contains the four methods SVM, SVM-RFE, Golub-Ratio and PAM, the second one is limited to SVM and SVM-RFE and the third one comprises PCA and PLS-DA. The performance of the signatures was determined based on the prediction outcomes obtained from six different classifiers which were evaluated in a 3-fold cross-validation. In the plot depicted above each bar corresponds to a certain classifier (see legend) and each group of bars refers to a certain ensemble of feature selection methods. The horizontal dashed line indicates the performance that would have been achieved by random guessing. The adjacent Venn diagrams illustrate informative genes common between signatures. **(B)** Same plots as in (A), but for C vs. NC classification after 14 days of repeated dosing. **(C)** Mean ROC scores and signature overlaps for GC vs. NGC classification after 3 days of treatment. **(D)** Same plots as in (C), but for GC vs. NGC classification after 14 days of repeated dosing.
(PDF)

Figure S4 **Selection frequency of signature genes.** The figure depicts a histogram showing the number of genes selected twice or more times as informative genes for compound class prediction. For the generation of this figure, we considered 8 predicted signatures for solving 4 binary classification tasks (C vs. NC, GC vs. NGC, GC vs. NC, NGC vs. NC) after 3 days and 14 days of repeated dosing, respectively. The genes selected 4 and 5 times, respectively, are listed next to the histogram.
(PDF)

Figure S5 **Expression profiles of most frequently selected signature genes.** The heatmap shows the expression profiles of all candidate biomarker genes which are contained in at least 3 of the 8 predicted signatures. These signatures were proposed for 4 different classification tasks (C vs. NC, GC vs. NGC, GC vs. NC, NGC vs. NC). For each classification task two treatment durations (3 days and 14 days) were considered, independently.
(PDF)

Table S1 mRNA signatures extracted with SVM, SVM-RFE, PAM and Golub-Ratio. The Excel sheets contain the Affymetrix IDs of the probesets selected by the following ensemble of feature selection methods: Support Vector Machines (SVM), SVM-based Recursive Feature Elimination (SVM-RFE), Prediction Analysis for Microarrays (PAM) and the statistical ratio proposed by Golub *et al.* (Golub-Ratio). If possible the corresponding gene symbol, EntrezGene ID and description are provided for each probeset.
(XLS)

Table S2 mRNA signatures extracted with PCA and PLS-DA. The Excel sheets contain the Affymetrix IDs of the probesets selected by the following ensemble of feature selection methods: Principal Component Analysis (PCA) and Partial Least Squares Discriminant Analysis (PLS-DA). If possible the corresponding gene symbol, EntrezGene ID and description are provided for each probeset.
(XLS)

Table S3 mRNA signatures extracted with SVM and SVM-RFE. The Excel sheets contain the Affymetrix IDs of the probesets selected by the following ensemble of feature selection methods: Support Vector Machines (SVM) and SVM-based Recursive Feature Elimination (SVM-RFE). If possible the corresponding gene symbol, EntrezGene ID and description are provided for each probeset.
(XLS)

Table S4 Published mRNA signatures for the detection of hepatocarcinogenicity in mice. The Excel sheets contain the Affymetrix IDs of published signatures for the toxicogenomics-based detection of carcinogens in mouse liver. If possible the corresponding gene symbol, EntrezGene ID and description are provided for each probeset. For signatures which were not derived from an Affymetrix platform, the original probeset IDs are listed in an additional column.
(XLS)
