# Supplementary material for: A Toxicogenomic Approach for the Prediction of Murine Hepatocarcinogenesis Using Ensemble Feature Selection
Source: PLoS One. 2013 Sep 10;8(9):e73938. doi: 10.1371/journal.pone.0073938 (PMC3769381; doi:10.1371/journal.pone.0073938)
Supplement: Figure S1 — Accuracy and stability of signatures for compound classification. (A) The line plots depict the mean performance of GC vs. NC classification after 3 days of repeated dosing, which was achieved based on gene sets extracted with different feature selection methods. Each curve corresponds to a feature selection method and the performance was assessed depending on the number of genes selected as informative features. The prediction accuracy was assessed on the samples left out from 25 random subsamplings of the dataset (bootstraps), each containing 90% of the data, and measured in terms of area under the ROC curve. The inset bar plot depicts the ROC scores averaged across bootstraps and signature sizes. (B) Performance of GC vs. NC classification after 14 days of treatment illustrated as in (A). (C) The correspondence of the extracted GC vs. NC gene sets across 25 bootstraps was assessed based on the Kuncheva stability index (KI) for each of the 4 employed feature selection methods. The KI was then for each method plotted against the number of selected signature genes. (D) Robustness of signatures for GC vs. NC classification after 14 days of treatment illustrated as in (C). (E, F) Prediction accuracy achieved with signatures for NGC vs. NC classification after (E) 3 days and (F) 14 days of repeated dosing, respectively, depicted as in (A). (G, H) Similar illustration as in (C) showing robustness of signatures for NGC vs. NC classification after (G) 3 days and (H) 14 days of administration, respectively. (PDF) [file pone.0073938.s001.pdf]

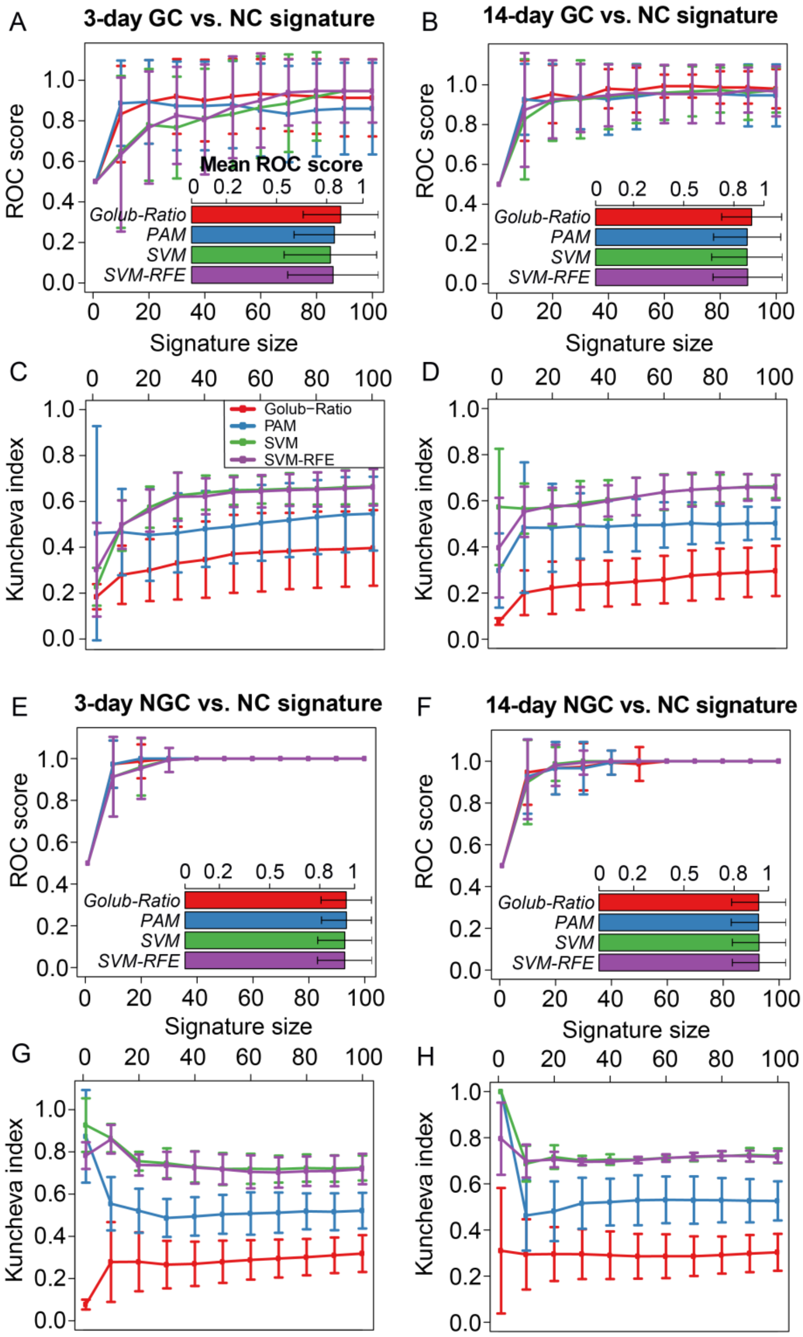

Figure S1: Accuracy and stability of signatures for compound classification

(A) The line plots depict the mean performance of GC vs. NC classification after 3 days of repeated dosing, which was achieved based on gene sets extracted with different feature selection methods. Each curve corresponds to a feature selection method and the performance was assessed depending on the number of genes selected as informative features. The prediction accuracy was assessed on the samples left out from 25 random subsamplings of the dataset (bootstraps), each containing 90% of the data, and measured in terms of area under the ROC curve. The inset bar plot depicts the ROC scores averaged across bootstraps and signature sizes. **(B)** Performance of GC vs. NC classification after 14 days of treatment illustrated as in (A). **(C)** The correspondence of the extracted GC vs. NC gene sets across 25 bootstraps was assessed based on the Kuncheva stability index (KI) for each of the 4 employed feature selection methods. The KI was then for each method plotted against the number of selected signature genes. **(D)** Robustness of signatures for GC vs. NC classification after 14 days of treatment illustrated as in (C). **(E, F)** Prediction accuracy achieved with signatures for NGC vs. NC classification after (E) 3 days and (F) 14 days of repeated dosing, respectively, depicted as in

(A). **(G, H)** Similar illustration as in (C) showing robustness of signatures for NGC vs. NC classification after (G) 3 days and (H) 14 days of administration, respectively.
