# Supplementary material for: A Toxicogenomic Approach for the Prediction of Murine Hepatocarcinogenesis Using Ensemble Feature Selection
Source: PLoS One. 2013 Sep 10;8(9):e73938. doi: 10.1371/journal.pone.0073938 (PMC3769381; doi:10.1371/journal.pone.0073938)
Supplement: Figure S2 — Classification results obtained for different signatures. (A) The four heatmaps show the predictions resulting from diverse binary classifiers for the discrimination of GC from NC after 3 days (left two heatmaps) or 14 days (right two heatmaps) of repeated dosing. For each dosing time two heatmaps are depicted which correspond to male and female mice, respectively. The rows correspond to different classifiers and the columns to different treatment groups. The continuous prediction scores, returned from the classifiers, were transformed to confidence scores between 0 and 1, which provide an estimate of the probability of class GC. The colorbar on top shows the true class annotation. The black vertical lines separate the test samples from the three folds of cross-validation. (B) Heatmaps illustrating the classification outcome of diverse predictors to distinguish NGC from NC based on characteristic gene expression profiles observed in male and female mouse liver samples after 3 or 14 days of administration. KNN, K-Nearest Neighbor; SVM, Support Vector Machine; PAM, Prediction Analysis for Microarrays. (PDF) [file pone.0073938.s002.pdf]

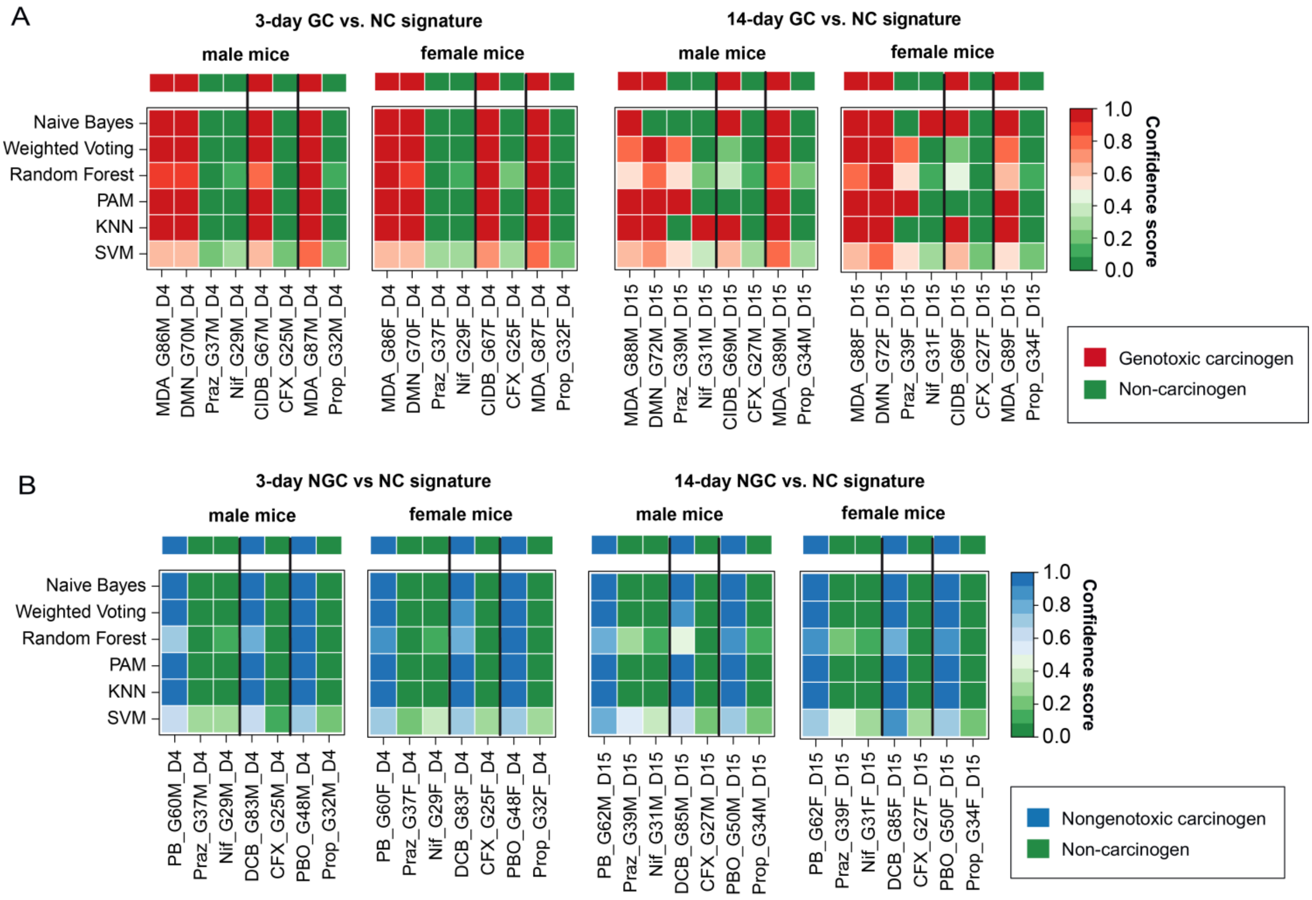

Figure S2: Classification results obtained for different signatures

(A) The four heatmaps show the predictions resulting from diverse binary classifiers for the discrimination of GC from NC after 3 days (left two heatmaps) or 14 days (right two heatmaps) of repeated dosing. For each dosing time two heatmaps are depicted which correspond to male and female mice, respectively. The rows correspond to different classifiers and the columns to different treatment groups. The continuous prediction scores, returned from the classifiers, were transformed to confidence scores between 0 and 1, which provide an estimate of the probability of class GC. The colorbar on top shows the true class annotation. The black vertical lines separate the test samples from the three folds of cross-validation. **(B)** Heatmaps illustrating the classification outcome of diverse predictors to distinguish NGC from NC based on characteristic gene expression profiles observed in male and female mouse liver samples after 3 or 14 days of administration. KNN, K-Nearest Neighbor; SVM, Support Vector Machine; PAM, Prediction Analysis for Microarrays.
