# Supplementary material for: A Toxicogenomic Approach for the Prediction of Murine Hepatocarcinogenesis Using Ensemble Feature Selection
Source: PLoS One. 2013 Sep 10;8(9):e73938. doi: 10.1371/journal.pone.0073938 (PMC3769381; doi:10.1371/journal.pone.0073938)
Supplement: Figure S3 — Classification results obtained for different ensembles of feature selection methods. (A) The grouped bar plots depict the area under the ROC curves achieved with signatures which were inferred based on three different ensembles of feature selection methods and applied for the separation of C and NC after 3 days of treatment. The first ensemble contains the four methods SVM, SVM-RFE, Golub-Ratio and PAM, the second one is limited to SVM and SVM-RFE and the third one comprises PCA and PLS-DA. The performance of the signatures was determined based on the prediction outcomes obtained from six different classifiers which were evaluated in a 3-fold cross-validation. In the plot depicted above each bar corresponds to a certain classifier (see legend) and each group of bars refers to a certain ensemble of feature selection methods. The horizontal dashed line indicates the performance that would have been achieved by random guessing. The adjacent Venn diagrams illustrate informative genes common between signatures. (B) Same plots as in (A), but for C vs. NC classification after 14 days of repeated dosing. (C) Mean ROC scores and signature overlaps for GC vs. NGC classification after 3 days of treatment. (D) Same plots as in (C), but for GC vs. NGC classification after 14 days of repeated dosing. (PDF) [file pone.0073938.s003.pdf]

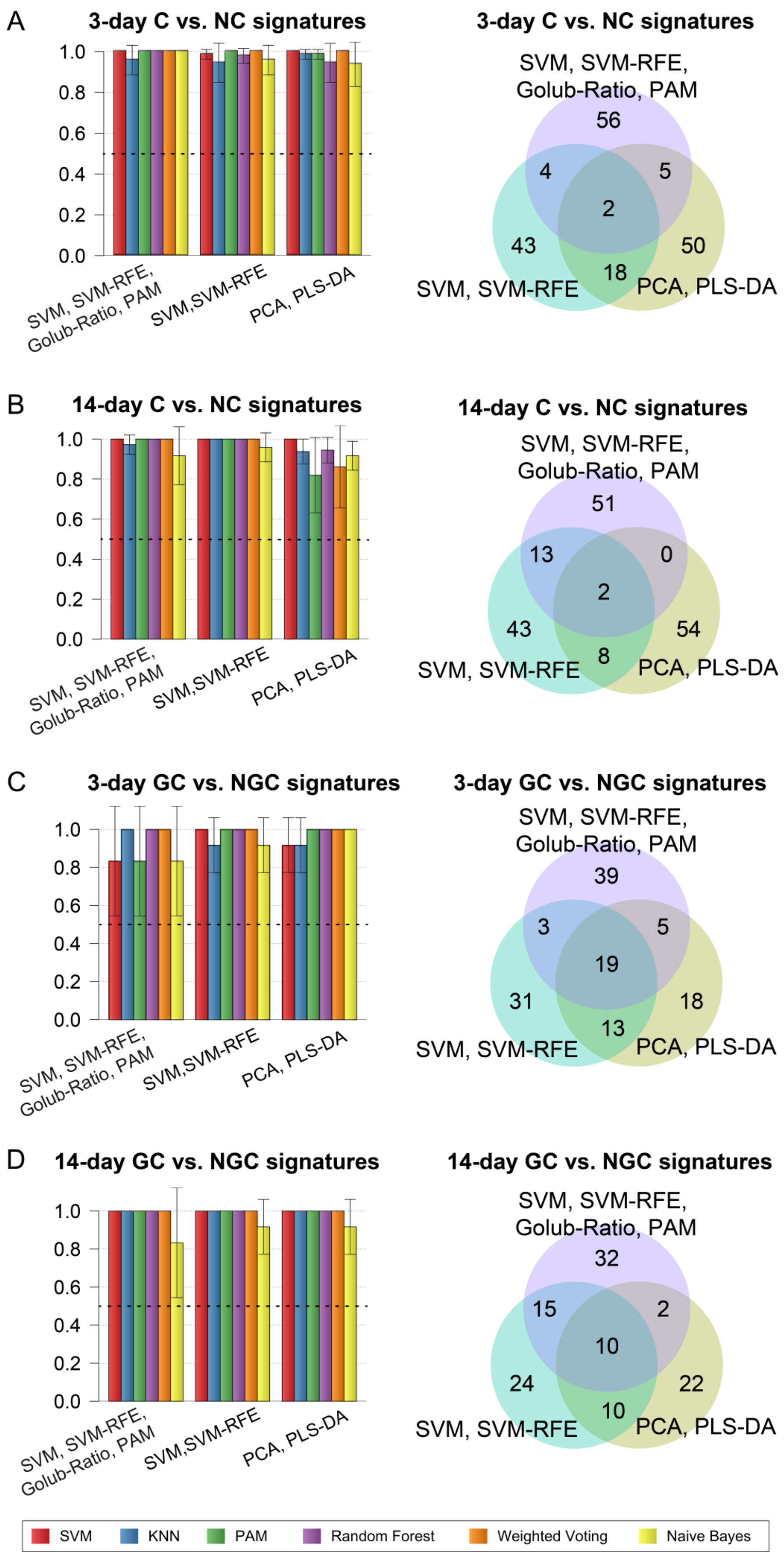

Figure S3: Classification results obtained for different ensembles of feature selection methods
(A) The grouped bar plots depict the area under the ROC curves achieved with signatures which were inferred based on three different ensembles of feature selection methods and applied for the

separation of C and NC after 3 days of treatment. The first ensemble contains the four methods SVM, SVM-RFE, Golub-Ratio and PAM, the second one is limited to SVM and SVM-RFE and the third one comprises PCA and PLS-DA. The performance of the signatures was determined based on the prediction outcomes obtained from six different classifiers which were evaluated in a 3-fold cross-validation. In the plot depicted above each bar corresponds to a certain classifier (see legend) and each group of bars refers to a certain ensemble of feature selection methods. The horizontal dashed line indicates the performance that would have been achieved by random guessing. The adjacent Venn diagrams illustrate informative genes common between signatures. **(B)** Same plots as in (A), but for C vs. NC classification after 14 days of repeated dosing. **(C)** Mean ROC scores and signature overlaps for GC vs. NGC classification after 3 days of treatment. **(D)** Same plots as in (C), but for GC vs. NGC classification after 14 days of repeated dosing.
