# Supplementary material for: A Toxicogenomic Approach for the Prediction of Murine Hepatocarcinogenesis Using Ensemble Feature Selection
Source: PLoS One. 2013 Sep 10;8(9):e73938. doi: 10.1371/journal.pone.0073938 (PMC3769381; doi:10.1371/journal.pone.0073938)
Supplement: Figure S4 — Selection frequency of signature genes. The figure depicts a histogram showing the number of genes selected twice or more times as informative genes for compound class prediction. For the generation of this figure, we considered 8 predicted signatures for solving 4 binary classification tasks (C vs. NC, GC vs. NGC, GC vs. NC, NGC vs. NC) after 3 days and 14 days of repeated dosing, respectively. The genes selected 4 and 5 times, respectively, are listed next to the histogram. (PDF) [file pone.0073938.s004.pdf]

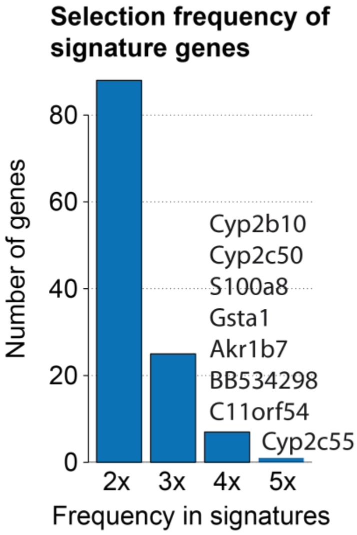

Figure S4: Selection frequency of signature genes

The figure depicts a histogram showing the number of genes selected twice or more times as informative genes for compound class prediction. For the generation of this figure, we considered 8 predicted signatures for solving 4 binary classification tasks (C vs. NC, GC vs. NGC, GC vs. NC, NGC vs. NC) after 3 days and 14 days of repeated dosing, respectively. The genes selected 4 and 5 times, respectively, are listed next to the histogram.
