# Supplementary material for: A Toxicogenomic Approach for the Prediction of Murine Hepatocarcinogenesis Using Ensemble Feature Selection
Source: PLoS One. 2013 Sep 10;8(9):e73938. doi: 10.1371/journal.pone.0073938 (PMC3769381; doi:10.1371/journal.pone.0073938)
Supplement: Figure S5 — Expression profiles of most frequently selected signature genes. The heatmap shows the expression profiles of all candidate biomarker genes which are contained in at least 3 of the 8 predicted signatures. These signatures were proposed for 4 different classification tasks (C vs. NC, GC vs. NGC, GC vs. NC, NGC vs. NC). For each classification task two treatment durations (3 days and 14 days) were considered, independently. (PDF) [file pone.0073938.s005.pdf]

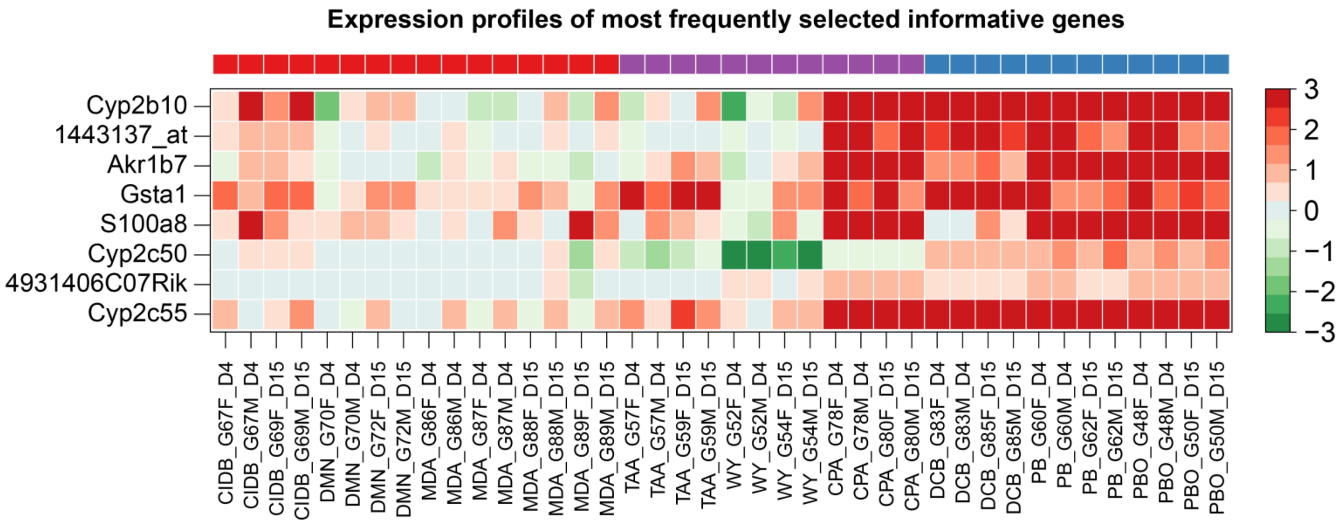

Figure S5: Expression profiles of most frequently selected signature genes
The heatmap shows the expression profiles of all candidate biomarker genes which are contained in at least 3 of the 8 predicted signatures. These signatures were proposed for 4 different classification tasks (C vs. NC, GC vs. NGC, GC vs. NC, NGC vs. NC). For each classification task two treatment durations (3 days and 14 days) were considered, independently.
